# Effect of water temperature and fish biomass on environmental DNA shedding, degradation, and size distribution

**DOI:** 10.1002/ece3.4802

**Published:** 2019-01-21

**Authors:** Toshiaki Jo, Hiroaki Murakami, Satoshi Yamamoto, Reiji Masuda, Toshifumi Minamoto

**Affiliations:** ^1^ Graduate School of Human Development and Environment Kobe University Kobe City Japan; ^2^ Maizuru Fisheries Research Station Kyoto University Kyoto Japan; ^3^ Department of Zoology, Graduate School of Science Kyoto University Kyoto Japan

**Keywords:** decay rate, environmental DNA, fish biomass, Japanese Jack Mackerel (*Trachurus japonicus*), quantitative real‐time PCR, shedding rate, size distribution, water temperature

## Abstract

Environmental DNA (eDNA) analysis has successfully detected organisms in various aquatic environments. However, there is little basic information on eDNA, including the eDNA shedding and degradation processes. This study focused on water temperature and fish biomass and showed that eDNA shedding, degradation, and size distribution varied depending on water temperature and fish biomass. The tank experiments consisted of four temperature levels and three fish biomass levels. The total eDNA and size‐fractioned eDNA from Japanese Jack Mackerels (*Trachurus japonicus*) were quantified before and after removing the fish. The results showed that the eDNA shedding rate increased at higher water temperature and larger fish biomass, and the eDNA decay rate also increased at higher temperature and fish biomass. In addition, the small‐sized eDNA fractions were proportionally larger at higher temperatures, and these proportions varied among fish biomass. After removing the fish from the tanks, the percentage of eDNA temporally decreased when the eDNA size fraction was >10 µm, while the smaller size fractions increased. These results have the potential to make the use of eDNA analysis more widespread in the future.

## 1 INTRODUCTION

Environmental DNA (eDNA) analysis is a new method that has been developed to improve the environmental management and assessment of aquatic ecosystems (Ficetola, Miaud, Pompanon, & Taberlet, 2008; Minamoto, Yamanaka, Takahara, Honjo, & Kawabata, 2012; Taberlet, Coissac, Hajibabaei, & Rieseberg, 2012; Thomsen & Willerslev, 2015). Environmental DNA, which is the DNA obtained directly from environmental samples such as water and sediments (Ficetola et al., 2008; Turner, Uy, & Everhart, 2015), is thought to derive from feces, mucus, skin, and gametes (Bylemans *et al*.,^2017^; Ficetola et al., 2008; Martellini, Payment, & Villemur, 2005; Merkes, McCalla, Jensen, Gaikowski, & Amberg, 2014). The presence of a target species can be estimated by detecting the eDNA in water samples instead of locating or capturing individuals (Lodge et al., 2012). These advantages have enabled noninvasive, quick, and wide‐ranging assessments of the presence/absence of species and their biodiversity and abundance in freshwater (Balasingham, Walter, Mandrak, & Heath, 2017; Bista et al., 2017; Deiner, Fronhofer, Mächler, Walser, & Altermatt, 2016; Fukumoto, Ushimaru, & Minamoto, 2015; Yamanaka & Minamoto, 2016) and marine environments (Boussarie et al., 2018; Lacoursière‐Roussel et al., 2018; Sigsgaard et al., 2016; Thomsen, Kielgast, Iversen, Møller, et al., 2012; Thomsen, Kielgast, Iversen, Wiuf, et al., 2012; Yamamoto et al., 2017).

Although various studies over the past decade have demonstrated successful eDNA detection, there is a lack of basic information about eDNA, such as its origin (i.e., the sources of eDNA), state, transport, and fate (Barnes & Turner, 2016; Hansen, Bekkevold, Clausen, & Nielsen, 2018). These factors affect the interpretation of eDNA monitoring. For example, the detectability and persistence of eDNA in environmental samples are mainly determined by eDNA shedding, transport, and degradation (Díaz‐Ferguson & Moyer, 2014; Goldberg, Strickler, & Pilliod, 2015; Strickler, Fremier, & Goldberg, 2015). Furthermore, various interactions between eDNA and its environment should also be taken into account (Barnes & Turner, 2016; Taberlet et al., 2012; Thomsen & Willerslev, 2015). To develop effective sampling methods and improve the reliability of this method, it is necessary to understand and accumulate basic information about eDNA. This study investigated the factors associated with eDNA shedding and degradation and the eDNA size distribution.

The degradation of eDNA mainly depends on (a) abiotic factors, such as water temperature (Strickler et al., 2015), pH (Tsuji, Yamanaka, & Minamoto, 2016), salinity (Dell'Anno & Corinaldesi, 2004), and ultraviolet (UV) radiation (Pilliod, Goldberg, Arkle, & Waits, 2014); (b) biotic factors, such as microbes and extracellular enzymes (Barnes et al., 2014); and (c) DNA characteristics, such as the differences between intra/extracellular DNA (Turner et al., 2014) and the length of the DNA fragments (Jo et al., 2017). In particular, water temperature seems to have a significant effect on eDNA; eDNA degradation was accelerated by higher temperature (Eichmiller, Best, & Sorensen, 2016; Lance et al., 2017; Strickler et al., 2015; Tsuji, Ushio, Sakurai, Minamoto, & Yamanaka, 2017). Furthermore, it is thought that water temperature does not directly affect eDNA degradation, such as the denaturation of double‐stranded DNA (Lindahl, 1993), but indirectly affects it through enzymatic hydrolysis by microbes and extracellular nucleases (Barnes & Turner, 2016; Levy‐Booth et al., 2007). It is likely that other factors also affect eDNA degradation by influencing the activity and abundance of microbes and extracellular nucleases. For example, the eDNA decay rate may vary depending on fish biomass because it is thought that higher fish biomass leads to increases in the abundance of bacteria in their local environment. However, there have been no studies on the relationship between the biomass of organisms and eDNA degradation.

The main factors associated with eDNA shedding are (1) the number and the biomass of organisms (Klymus, Richter, Chapman, & Paukert, 2015; Takahara, Minamoto, Yamanaka, Doi, & Kawabata, 2012); (2) the developmental stage of the organisms (Maruyama, Nakamura, Yamanaka, Kondoh, & Minamoto, 2014); (3) the behavior of organisms (Dunn, Priestley, Herraiz, Arnold, & Savolainen, 2017); and (4) the stress against organisms (Bylemans, Furlan, Gleeson, Hardy, & Duncan, 2018; Pilliod et al., 2014). In addition, considering that feed intake increased the eDNA shedding (Klymus et al., 2015), eDNA shedding rate is likely to depend on (5) the metabolism and physiological activity of the organisms. For example, water temperature plays an important role in the growth and metabolism of fish (Clarke & Johnston, 1999; Morita, Fukuwaka, Tanimata, & Yamamura, 2010; Sandersfeld, Mark, & Knust, 2017). For juvenile European sea bass (*Dicentrarchus labrax*), feed intake (FI) and efficiency (FE) and total ammonia nitrogen (TAN) excretion increased at 25°C, which was the optimum temperature for the growth of this species (Person‐Le Ruyet, Mahe, Le Bayon, & Le Delliou, 2004). Therefore, it is likely that eDNA shedding increases at the optimum temperature for fish growth. However, there have been no studies on the relationship between temperature and eDNA shedding.

Although the physiological origins of the material collected as eDNA remain uncertain (Barnes & Turner, 2016), previous studies have shown that eDNA size varied between >180 and <0.2 μm, and the most abundant eDNA size range for macro‐organisms was from 1 to 10 μm (Sassoubre, Yamahara, Gardner, Block, & Boehm, 2016; Turner et al., 2014; Wilcox, McKelvey, Young, Lowe, & Schwartz, 2015). The different eDNA sizes reflect the various eDNA states (e.g., intra/extracellular DNA and within live/dead cells). Therefore, eDNA persistence and degradation could vary depending on the eDNA states. For example, eDNA size distribution might vary depending on the temperature of the rearing water and the biomass of organisms and may also temporally vary after removal of the organisms.

The aim of this study was to determine the effect of water temperature and fish biomass on eDNA shedding, degradation, and size distribution, and to refine the eDNA analysis method. We used Japanese Jack Mackerel (*Trachurus japonicus*) as a target species due to its use in previous eDNA studies (Jo et al., 2017; Yamamoto et al., 2017, 2016) and due to its economic importance as one of the most consumed fish species in Japan. It is therefore critical to understand and accumulate such basic information on eDNA for this species.

## 2 MATERIALS AND METHODS

### 2.1 Tank experiment

#### 2.1.1 Experimental design

The experiments took place at the Maizuru Fisheries Research Station of Kyoto University, Japan, which is in front of Maizuru Bay, from June 2016 to July 2017 (Supporting Information Table S1). Acrylic 200‐L tanks were assigned four water temperatures (13, 18, 23, and 28°C) and three fish biomass levels (Small, Medium, and Large; see below for fish size details), which resulted in twelve treatment levels in this study. Four temperature levels were selected based on the preference temperature of our target fish (i.e., around 20°C; Nakamura & Hamano, 2009) and within the range of bottom water temperature when this species is recorded at the sampling site (Masuda, 2008). Four tank replicates were prepared for each treatment level. Two experimental tanks were placed in each water bath and heated using a 100 V 1.0 kW heater (Mitsubishi, Japan). The temperature was regulated using a thermostat (Nitto, Derthermo, Japan). The tanks were kept at a constant water temperature throughout the experiment and were aerated using a pump. The water temperature was measured every morning using a digital thermometer (Tetra, Spectrum Brands Japan; Supporting Information Table S1). Filtered seawater used in the experiment was pumped from 6 m depth off the Research Station where the water quality is scarcely impacted by rainfall and other environmental factors. Before use, it was filtered by passing through five different materials starting with coarse polyvinyl fabric (Saranlock OM‐150, Asahi Kasei, Japan) and ending with fine sand of around 0.6 mm in diameter (5G‐ST, Nikkiso Eiko, Japan). Inlet water was poured at a rate of 600 ml/min into each tank.

After the experimental tanks had been prepared, three Japanese Jack Mackerels were added to each tank and they were left in the tank for about 1 week prior to the experiments for the acclimation (Sassoubre et al., 2016; Takahara et al., 2012). For the tank experiment using Medium‐sized fish, all Japanese Jack Mackerels were used only once. On the other hand, for the tank experiment using Large‐ and Small‐sized fish, some of the fish were used more than once (the details were seen in Supporting Information Table S1). In these experimental periods, we repeatedly used all the fish which had survived, and supplied replacements for the dead or dying fish. The fish were fed a small amount of krill every morning until the day before water sampling. The bottom of each tank was cleaned an hour after feeding to eliminate the effect of the feces, and, on the sampling day, the fish were starved. After 1 week, the Japanese Jack Mackerels were quickly removed from each tank, and their total length (TL) and wet weight were measured. A water sample from each tank was also collected (water sampling details are described below). The TLs and wet weights for each fish biomass level were 6.2 ± 0.4 cm and 2.3 ± 0.5 g (Small), 11.7 ± 1.2 cm and 13.4 ± 4.2 g (Medium), and 21.4 ± 3.1 cm and 106.5 ± 48.4 g (Large; both mean ±1 *SD*; Supporting Information Table S2). There were no significant differences in TLs and wet weights among fish within each fish size group (ANOVA, *p* > 0.1).

#### 2.1.2 eDNA sampling

The eDNA was sampled using two different methods. The first method used a 47‐mm‐diameter glass microfiber filter GF/F (nominal pore size 0.7 μm; GE Healthcare Life Science, Little Chalfont, UK) to estimate eDNA shedding and decay rates, and the second method used a series of 47‐mm‐diameter polycarbonate membrane filters (pore size 10, 3, 0.8, and 0.4 or 0.2 μm; MILLIPORE, US) to estimate eDNA size distribution. Disposable gloves were worn when collecting water samples, and the outside of the sampling bottles was washed with tap water after the samples were collected. This was to prevent contamination during water sampling and filtration. The filtering devices (i.e., filter funnels [Magnetic Filter Funnel, 500 ml capacity; Pall Corporation, Westborough, MA, USA], plastic holders [ADVANTEC, Japan], nipple joints [ADVANTEC, Japan], hoses [TOYOX, Japan], 1‐L beakers, tweezers, and sampling bottles used for water sampling) were bleached after every use in 0.1% sodium hypochlorite solution for at least 5 min.

##### eDNA sampling (i): estimation of eDNA shedding and decay rates

The aim of this sampling was to estimate Japanese Jack Mackerel eDNA shedding and decay rates and to investigate how they were affected by water temperature and fish biomass. The time just after removing the fish from each tank was defined as time 0, and more than 1 L of water was collected from each tank at 0, 2, 4, 8, 16, 24, 48, 72, and 96 hr (these time points are referred as time 0 to time 96). An additional water sample was collected at 120 and 216 hr after time 0 (i.e., time 120 and 216) in the tank experiments containing Medium‐sized fish to measure eDNA persistence in the tank, which was the first experimental period in our overall study (see Supporting Information Tables S1 and S4). Water samples were also collected the day before removing the fish from the tanks to measure the eDNA concentrations at a steady state. This was defined as time before fish removal (i.e., time bfr). The term “steady state” was defined as being when eDNA shedding was in equilibrium with total eDNA degradation and dilution in each tank after the eDNA concentration had stabilized (Sansom & Sassoubre, 2017; Sassoubre et al., 2016).

After water collection, the 1 L water samples were immediately filtered with a GF/F filter. At each sampling time, 1 L of distilled water was also filtered as a filtration negative control. Furthermore, 1 L of inlet water was sampled from each tank at time 24 to evaluate the background Japanese Jack Mackerel eDNA concentration in the inlet water. Note that the experimental tanks were flow‐through until removing the fish from the tank, while we stopped inlet water once fish were removed. All filter samples were kept at −20°C after filtration until needed for eDNA extraction.

##### eDNA sampling (ii): estimation of eDNA size distribution

The aim in this sampling was to estimate the eDNA size distribution for Japanese Jack Mackerels and to investigate the effect of temperature, fish biomass, and the time passage on eDNA size distribution. Sequential filtration was performed using a combination of plastic holders, nipple joints, and hoses. The water samples were 500 ml in volume, and they were filtered using four polycarbonate membrane filters (except for the Large fish biomass level in the 28°C treatment where 250 ml water samples were taken due to filter clogging). At each sampling time, 500 ml of distilled water was also sequentially filtered as a filtration negative control.

For all fish biomass levels, the water samples were collected at time bfr using a series of polycarbonate membrane filters with 10, 3, 0.8, and 0.4 μm pore sizes. For the Small and Large fish biomass tank experiments, the water samples were also collected at time bfr, 0, 6, 12, and 18 using the same filters with 10, 3, 0.8, and 0.2 μm pore sizes (Supporting Information Figure S1). All filter samples were kept at −20°C until eDNA extraction.

#### 2.1.3 DNA extraction

The total eDNA on each filter was extracted using a DNeasy Blood and Tissue Kit (Qiagen, Hilden, Germany), and all eDNA extracts were placed in a freezer (−20°C) until quantitative PCR analysis. The DNA was extracted from the GF/F filters by a method used in a previous study (Jo et al., 2017). Briefly, a filter sample was placed in the suspended part of a Salivette tube (Sarstedt, Nümbrecht, Germany). Then, 420 μl of a solution containing 20 μl proteinase K, 200 μl buffer AL, and 200 μl pure water was placed on the filter and the tube was incubated at 56°C for 30 min. After incubation, the liquid held in the filter was collected by centrifugation at 5,000 *g* for 3 min. To increase the eDNA yield, the filter was re‐washed with 200 μl TE buffer for 1 min and the liquid was again collected after centrifugation at 5,000 *g* for 3 min. Then, 500 μl ethanol was added to the collected liquid and the mixture transferred to a spin column. Subsequently, the total eDNA was eluted in 100 μl AE buffer following the manufacturer's instructions.

The DNA was extracted from the polycarbonate membrane filters using a DNeasy Blood & Tissue Kit with slight modifications to its protocol (Matsuhashi *et al*., unpublished). Briefly, tweezers were used to place a filter sample in a spin column. Then, 320 μl of a solution containing 20 μl proteinase K, 150 μl buffer AL, and 150 μl TE buffer was added to the sample and the mixture was incubated it at 56°C for 30 min. After incubation, 150 μl ethanol was added to the filter sample, and the mixture centrifuged in a spin column at 6,000 *g* for 1 min. To increase the eDNA yield, the filter was re‐washed with a 300 μl solution that contained 100 μl TE buffer, 100 μl buffer AL, and 100 μl ethanol, for 1 min, and then, the mixture was centrifuged 6,000 *g* for 1 min. The sample filter was removed from the spin column, and the total eDNA was eluted in 100 μl AE buffer following the manufacturer's instructions.

#### 2.1.4 Quantification of eDNA using qPCR

The amount of eDNA derived from Japanese Jack Mackerel at each time point was evaluated by quantifying the CytB gene copy numbers using real‐time TaqMan PCR and the StepOnePlus Real‐Time PCR system (Applied Biosystems, Foster City, CA, USA). The primer/probe set in this study specifically amplified the Japanese Jack Mackerel DNA and targeted a 127‐bp fragment of the mitochondrial CytB gene (Yamamoto et al., 2016). The number of Japanese Jack Mackerel CytB genes in each 2 μl eDNA solution sample was quantified by simultaneously performing qPCR using a dilution series of standards containing 3 × 10^1^–3 × 10^4^ copies of a linearized plasmid that contained synthesized artificial DNA fragments of the full CytB gene sequence for Japanese Jack Mackerel (Jo et al., 2017). In addition, a 2 μl pure water sample was analyzed as a PCR‐negative control. Each 20 μl TaqMan reaction contained 2 μl DNA extract, a final concentration of 900 nM of forward and reverse primers, and 125 nM of TaqMan probe in 1× TaqMan Gene Expression PCR Master Mix. Quantitative PCR was performed with the following conditions: 2 min at 50°C, 10 min at 95°C, 55 cycles of 15 s at 95°C, and 1 min at 60°C. All the qPCRs for eDNA extracts, standards, and negative controls were performed in triplicate. The DNA concentrations in the water samples were calculated by averaging the triplicate. All positive replicates were treated as having been successfully quantified (i.e., no “limit of quantification” was set) following the previous studies not setting the limit of quantification (Minamoto et al., 2017; Pilliod et al., 2014; Thomsen, Kielgast, Iversen, Møller, et al., 2012; Thomsen, Kielgast, Iversen, Wiuf, et al., 2012). Each replicate showing non‐detection (PCR‐negative) was regarded as containing 0 copies (Ellison, English, Burns, & Keer, 2006). We did not test PCR inhibition in all PCR runs because it is unlikely that PCR inhibition occurred using the water samples derived filtered seawater (Yamamoto et al., 2017, 2016).

### 2.2 Data analysis

R version 3.2.4 (R Core Team, 2016) was used to perform the statistical analyses. One of the tanks containing Large fish at 28°C was excluded from the statistical analysis due to fish mortality. The statistical analyses are in detail described in the sections below.

#### 2.2.1 Environmental DNA shedding and decay rates

The Japanese Jack Mackerel eDNA decay rates were estimated from the eDNA decay curves obtained from each experimental tank. Previous studies have estimated eDNA decay rates by fitting an exponential decay model (Eichmiller et al., 2016; Minamoto et al., 2017; Sansom & Sassoubre, 2017; Sassoubre et al., 2016; Thomsen, Kielgast, Iversen, Møller, et al., 2012; Thomsen, Kielgast, Iversen, Wiuf, et al., 2012; Tsuji et al., 2017) as follows:

$$C_{t}=C_{0}e^{-k*t}\left(\text{model}\;1\right)$$

where *C_t_* is the eDNA concentration at time *t* (copies/L), *C*_0_ is the eDNA concentration at time 0, and *k* is the decay rate constant (/hour). After referring to Tsuji et al. (2017), model 1 was extended to include the effect of water temperature and/or fish biomass in the tank. The fitness of each regression model was then compared. These models were as follows:

$$C_{t}=C_{0}e^{-\left(b*T+a\right)*t}\left(\text{model}\;2\right)$$

$$C_{t}=C_{0}e^{-\left(c*D+a\right)*t}\;\left(\text{model}\;3\right)$$

$$C_{t}=C_{0}e^{-\left(b*T+c*D+a\right)*t}\;\left(\text{model}\;4\right)$$

where *T* is the water temperature (°C), *D* is the total wet weight of Japanese Jack Mackerel in each 200 L tank (g/200 L), and *a*, *b,* and *c* are constants, which were estimated by analyzing the nonlinear least‐squares regression of the nls function in R. The eDNA concentrations at each time point were adjusted by the eDNA concentration at time 0 (i.e., *C*_0_ in each tank was regarded as 1), and the total wet weight of Japanese Jack Mackerel was log‐transformed. The effects of water temperature and fish biomass on the eDNA decay rate were investigated by comparing the four models using Akaike's Information Criterion (AIC), and the model with the smallest AIC values was accepted as the most supported model. The estimated parameters of this model were used to calculate the eDNA decay rates at each treatment level.

Methods used in previous studies (Maruyama et al., 2014; Sansom & Sassoubre, 2017; Sassoubre et al., 2016), with some modifications, were used to estimate Japanese Jack Mackerel eDNA shedding rates per tank. This is expressed using the following equation:

$$S=\left(k+R/V\right)\times C_{\text{cons.}}\times V$$

where *S* is the eDNA shedding rate in each tank (copies/hour), *k* is the estimated eDNA decay rate in each tank (/hour; see above), *C*_cons._ is the eDNA concentration at a steady state (i.e., at time –24; copies/L), *R* is the flow rate of the inlet water (L/hr), and *V* is the volume of the experimental tanks (L). Therefore, *R/V* is the dilution rate in the experimental tanks (/hour). This equation is derived from an ordinary differential equation representing the change in the abundance of eDNA with time as follows:

$$V\frac{\mathrm{dC}}{\mathrm{dt}}=S-\beta CV$$

Briefly, at steady state (i.e., time before fish removal), we assume that eDNA shedding was in equilibrium with total eDNA degradation and dilution (i.e., *β* = *k* + *R/V*) in each tank. Thus, $\frac{\mathrm{dC}}{\mathrm{dt}}=0$ and *S* = *βCV* = (*k* + *R/V*) × *C*_cons._ × *V*. The eDNA shedding rate per fish body weight (copies/hr/g) was estimated by dividing the eDNA shedding rates per tank by the total wet weight of the fish in the tank. These shedding rates were log‐transformed, and a two‐way ANOVA and a posthoc Tukey–Kramer test were performed to investigate the effects of water temperature, fish size, and their interaction.

#### 2.2.2 Environmental DNA size distribution

The eDNA concentrations in each size fraction were converted to a percentage of total sequential filtration (%). The percentage of eDNA calculated above was arcsin transformed to reduce skewness and to meet the normality criteria (Cook & Heyse, 2000). Any eDNA particles smaller than 0.4 or 0.2 μm were not assessed because the amount of eDNA in this size fraction seemed to be very small (Turner et al., 2014).

First, the samples that had passed through a sequential filter with 10, 3, 0.8, and 0.4 μm pore sizes at time bfr were used to verify the effect of water temperature and fish biomass on eDNA size distribution at the steady state. We calculated the Spearman's rank correlation coefficients between the percentage of eDNA and water temperature at each size fraction, where total fish biomass levels were not considered (i.e., we did not analyze these correlations at each fish biomass level). In addition, we performed a one‐way ANOVA and a posthoc Tukey–Kramer test to verify the difference of the percentage of eDNA among fish biomass levels at each size fraction, where temperature levels were not considered (i.e., we did not analyze these tests at each temperature level).

Second, the samples that had passed through a sequential filter with 10, 3, 0.8, and 0.2 μm pore sizes at times bfr to 18 were used to compare the eDNA size distribution at each time point. We performed a Wilcoxon's rank sum test between the percentage of eDNA before and after removing the fish from the tanks (i.e., time bfr vs. time 0) at each size fraction. In addition, we calculated the Spearman's rank correlation coefficients between the percentage of eDNA and time point (time 0–18) at each size fraction. For these analyses, all fish biomass and temperature levels were put together. We hypothesized that (a) eDNA size distribution would change before and after the fish removal because the handling stress might lead the fish to shed more DNA; and (b) eDNA size distribution would temporally change after the fish removal because the persistence of eDNA might vary depending on the state and size of eDNA.

## 3 RESULTS

In all the qPCR runs, the *R* ^2^ values, slope, Y‐intercept, and PCR efficiency of the calibration curves were 0.994 ± 0.004, −3.467 ± 0.101, 42.650 ± 0.852, and 94.410 ± 3.821, respectively (mean ±1 *SD*; Supporting Information Table S3). The amplification of target eDNA was seen in some of inlet water samples and in the filtration negative controls. This means that some contamination was mainly derived from the process of water filtering. However, these copy numbers were much lower than in the samples taken from the experimental tanks (the detail was seen in Supporting Information Tables S4 and S5). Therefore, the Japanese Jack Mackerel eDNA in the inlet water and low‐level cross‐contamination among samples is not likely to have affected our results.

### 3.1 Effect of water temperature and fish biomass on eDNA shedding and decay rates

**Figure 1:**
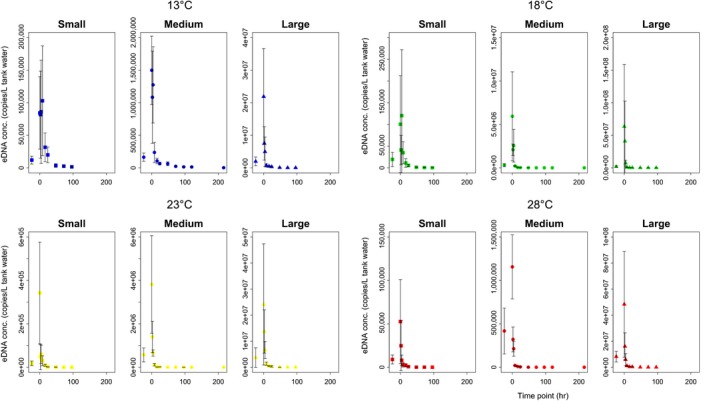
Decay curves for Japanese Jack Mackerel eDNA in the experimental tanks. Dots show eDNA concentrations per liter of tank water at each time point (Small: square, Medium: circle, Large: triangle; average of four tank replicates, except for the Large at 28°C). Error bars show the standard deviations (*SD*)

The eDNA concentration at time 0 (when the fish were removed) increased by 10–100 times compared to the steady state (i.e., time bfr), which could be due to the handling stress when removing the fish. After removal, the eDNA concentration decreased exponentially (Figure 1). This tendency was consistently observed in all treatments (Figure 1).

**Figure 2:**
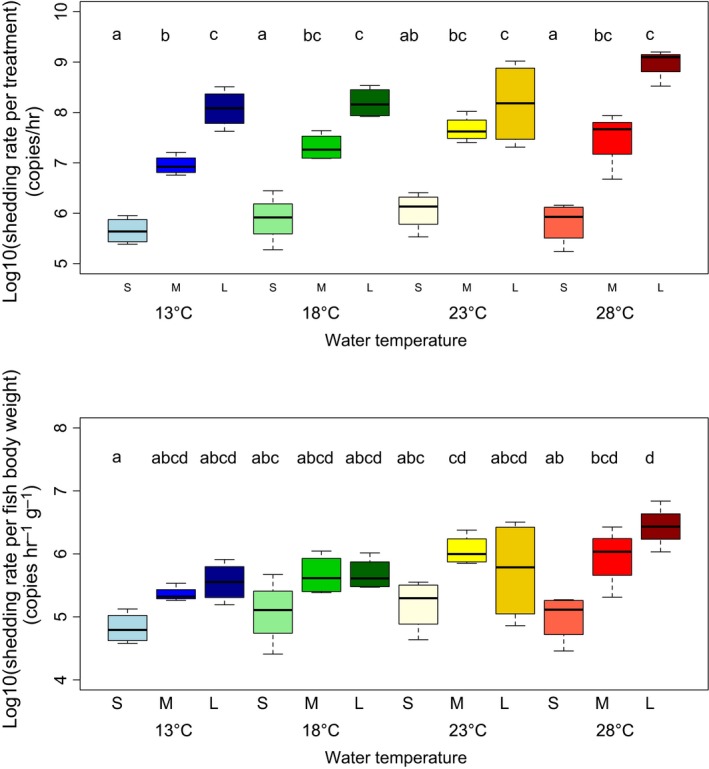
Results for eDNA shedding rate per treatment (upper) and per fish body weight (lower). Both boxplots show the comparison of eDNA shedding rates among four temperature and three biomass levels (average of four tank replicates, except for the Large size at 28°C). Factor levels with different letters are statistically significantly different (*p* < 0.05) based on posthoc Tukey–Kramer tests.

The most supported model for the eDNA decay curves based on AIC values was model 4, which included both water temperature and fish biomass in the tank as explanatory variables (Table 1; *C_t_* = *C*_0_ *e* ^−(^ *^b^* ^*^ *^T^* ^+^ *^c^* ^*^ *^D^* ^+^ *^a^* ^)*^ *^t^*). The eDNA decay rates for each treatment level were calculated based on these parameters, and the results showed that Japanese Jack Mackerel eDNA decay increased as the temperature and fish biomass in the experimental tanks rose (Table 2). The two‐way ANOVA and posthoc Tukey–Kramer test results showed that both fish biomass and temperature significantly affected eDNA shedding rates per each treatment (*p* < 0.05; Figure 2), and both partly affected eDNA shedding rates per fish body weight (*p* < 0.05). Their interaction was not significant (*p* > 0.1).

**Table 1:** The eDNA decay curve model results for the tank experiments estimated by the nls function in R

Model	*C*_0_	*b*	*c*	*a*	AIC	ΔAIC
*C*_(_ *_t_*_)_ = *C*_0_*exp(b*t)	0.9590***	−0.1876***	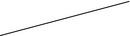	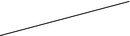	70.5144	127.4962
*C*_(_ *_t_*_)_ = *C*_0_*exp{(b*T + *a*)*t}	0.9737***	−0.0176***	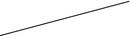	0.1415***	6.1045	63.0863
*C*_(_ *_t_*_)_ = *C*_0_*exp{(c*D + *a*)*t}	0.9455***	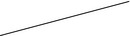	−0.1004***	−0.0260	48.9897	105.9715
*C*_(_*_t_*_)_ = *C*_0_*exp{(b*T + c*D + *a*)*t}	1.0029***	−0.0173***	−0.1027***	0.2732***	**−56.9818**	**0.0000**

Note

The AIC values (bold) were used to identify the most supported model for the eDNA decay curves. Asterisks show the significant effects (*p* < 0.001) of each parameter. The best model included both water temperature (*T*) and fish density (*D*, log‐transformed) as explanatory variables, which indicated that both water temperature and fish density influence eDNA degradation

**Table 2:** Japanese Jack Mackerel eDNA decay rate results when estimated by the best model. Values for eDNA decay rates are the mean ±1 *SD* (average of four tank replicates, except for the Large size at 28°C). Note that the treatment of 28°C ‐ Large fish biomass level had only three tank replicates due to fish mortality

eDNA decay rates (/hour)			
	Small	Medium	Large
13°C	0.0372 ± 0.0028	0.1154 ± 0.0077	0.2110 ± 0.0061
18°C	0.1219 ± 0.0049	0.2074 ± 0.0047	0.2969 ± 0.0077
23°C	0.2126 ± 0.0052	0.2903 ± 0.0049	0.3753 ± 0.0051
28°C	0.2959 ± 0.0052	0.3689 ± 0.0115	0.4686 ± 0.0126

### 3.2 Effect of water temperature and fish biomass on eDNA size distribution

**Figure 3:**
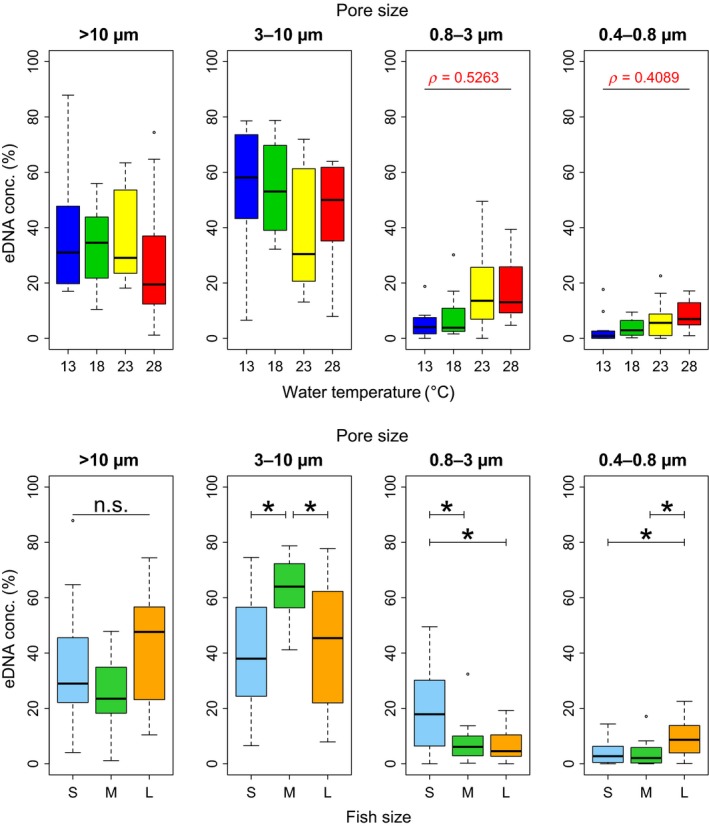
Results for eDNA size distributions at the steady state. Upper boxplots show a comparison between the four water temperature levels (13°C, 18°C, 23°C, and 28°C) when all fish biomass levels (Small, Medium, and Large) are combined. The lower boxplots show comparisons between the three fish biomass levels when all water temperature levels are combined. Stars show significant differences in the percentage of eDNA for each fish biomass level based on posthoc Tukey–Kramer tests. Only significant correlations (*p* < 0.05) are shown in the boxplots

Japanese Jack Mackerel eDNA size distribution at the steady state varied depending on water temperature and fish biomass. The 0.8–3 μm and 0.4–0.8 μm eDNA size proportions showed significant positive correlations with water temperature (*p* < 0.01; Figure 3), while there were no significant correlations between the percentage of eDNA and water temperature at >10 µm and 3–10 µm size fraction (*p* > 0.05). Each eDNA size fraction, except for the >10 μm size fraction, was significantly affected by the three different fish biomass levels. The highest eDNA proportion was 3–10 μm for the Medium fish size (*p* < 0.05), whereas it was 0.8–3 μm for the Small fish size (*p* < 0.05), and 0.4–0.8 μm for the Large fish size (*p* < 0.01; Figure 3). The difference of the percentage of eDNA at >10 μm size fraction was not significant but marginal among the three fish biomass levels (*p* = 0.0862), and the mean >10 μm eDNA proportion was highest for the Large fish size (Figure 3).

### 3.3 Temporal dynamics of eDNA size distribution

**Figure 4:**
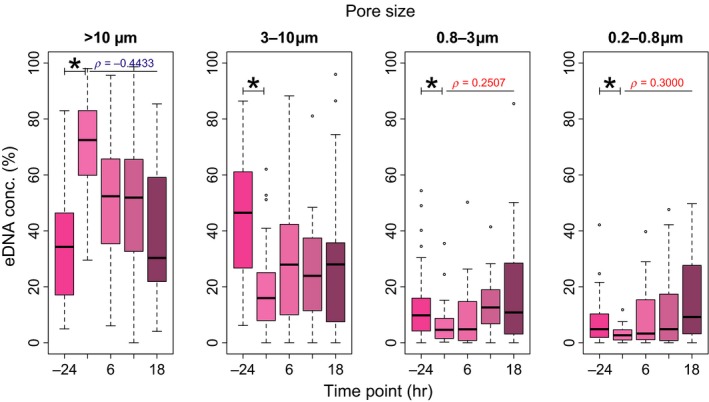
Temporal dynamics results for eDNA size distribution from time bfr (bright pink) to 18 (dark pink). Sum of the same colors at each pore size gives 100%. Boxplots show the temporal dynamics for the different Japanese Jack Mackerel eDNA percentages at each pore size when all water temperature levels (13°C, 18°C, 23°C, and 28°C) and fish biomass levels (Small, Medium, and Large) are combined. The figure “‐24” below means the time bfr. Stars show significant differences (*p* < 0.05) in the eDNA concentration proportions between time bfr and 0. Only significant correlations (positive in red and negative in blue) from time 0 to 18 are shown in the boxplots

The Japanese Jack Mackerel eDNA size distribution temporal change varied considerably. At the steady state (i.e., time bfr), most of the eDNA was in the 3–10 μm size fraction. Just after removing the fish from the tanks (i.e., time 0), the percentage of eDNA in the >10 μm size fraction increased considerably, whereas the percentages of eDNA at other size fractions decreased (Figure 4). Between time bfr and 0, there were significant differences of the percentage of eDNA at all size fraction (*p* < 0.05; Figure 4). After time 0, the percentage of eDNA in the >10 μm size fraction was significantly negatively correlated with sampling time (*ρ* = −0.4433, *p* < 0.0001), whereas the percentages of eDNA in the 0.8–3 μm and 0.2–0.8 μm size fractions were significantly positively correlated with sampling time (*ρ* = 0.2507, *p* < 0.01; *ρ* = 0.3000, *p* < 0.001, respectively). There was no significant correlation between the percentage of eDNA at the 3–10 μm size fraction and sampling time (*p* = 0.3297).

## 4 DISCUSSION

### 4.1 Factors affecting the degradation of eDNA

The regression analysis results showed that higher water temperatures and higher fish biomass accelerated eDNA degradation (Tables 1 and 2). These results supported previous studies that had also shown water temperature‐dependent degradation of eDNA (Eichmiller et al., 2016; Lance et al., 2017; Strickler et al., 2015; Tsuji et al., 2017). However, this is the first study to show that eDNA degradation is associated with fish biomass. It is also the first to show the water temperature‐dependent degradation of marine fish eDNA. As moderately higher temperatures (<50°C) stimulate microbial metabolism and exonuclease activity (Corinaldesi, Beolchini, & Dell'Anno, 2008; Poté, Ackermann, & Wildi, 2009), and high fish density can lead to the increase in microbial activity (Barnes et al., 2014; Bylemans et al., 2018), these results are likely to support the hypothesis that the activity and abundance of microbes and extracellular nucleases significantly affect eDNA degradation (Barnes & Turner, 2016; Levy‐Booth et al., 2007; Nielsen, Johnsen, Bensasson, & Daffonchio, 2007).

Several previous studies on eDNA decay rates addressed the persistence and degradation of marine fish eDNA. Sassoubre et al. (2016) had a similar experimental design to this study and targeted marine fish (Northern Anchovy [*Engraulis mordax*], Pacific Sardine [*Sardinops sagax*], and Pacific Chub Mackerel [*Scomber japonicus*]). They reported that eDNA decay rates were 0.055–0.101 (/hour), which was within the range reported by our study (0.035–0.485 [/hour]). The wider decay rate range in our study may be due to the effect of fish biomass in experimental tanks. In Sassoubre et al. (2016), the density of three marine fish ranged from 0.2 to 2.0 g/L, whereas the range was from 0.03 to 2.3 g/L in ours. It would be common that fish biomass affects eDNA degradation in seawater. Further study would be needed to reveal the relationship between the abundance/biomass of organisms and eDNA concentrations.

### 4.2 Factors affecting the shedding of eDNA

The eDNA shedding rates varied according to fish biomass (Figure 2), which supports previous studies (Doi et al., 2016, 2015; Klymus et al., 2015). It was not expected that the eDNA shedding rates per fish body weight at some temperature levels were also positively correlated with fish biomass (Figure 2), as the surface area per fish body weight was negatively correlated with fish body weight (Bergmann, 1847). One explanation could be the excessive effect of fish density in tanks, particularly for the Large fish biomass level. For example, the fish might have touched each other more often or rubbed up against the net in the tanks.

Our study demonstrated that eDNA shedding rate depended on water temperature (Figure 2). Some studies have shown that eDNA concentration did not depend on water temperature (Klymus et al., 2015; Takahara et al., 2012). However, they did not estimate the true eDNA shedding rate (i.e., they estimated the accumulated amount of eDNA) and thus could not divide the effects of eDNA shedding and degradation. It is important to investigate how water temperature influences not only the amount of eDNA detected in the field but also the eDNA shedding rate. As mentioned above, the metabolism of fish greatly depends on water temperature (Morita et al., 2010; Person‐Le Ruyet et al., 2004), which means that high water temperatures can be stressful for fish (Barton, 2002; Takahara et al., 2014). Therefore, Japanese Jack Mackerel eDNA shedding rate would be expected to increase at around 20°C, which is the optimal temperature for this species (Nakamura & Hamano, 2009) or at 28°C, which was the highest water temperature in these experiments. The results showed that both eDNA shedding rates per each treatment and per fish body weight tended to increase at higher temperatures, which confirmed the above expectations.

### 4.3 Environmental DNA size distribution

The results showed that the percentage of eDNA at the 0.8–3 μm and 0.4–0.8 μm size fractions increased with higher water temperatures (Figure 3). As the primer/probe sets in this study targeted mitochondrial DNA, the eDNA detected at these small size fractions was considered to be mainly mitochondria itself (0.5–2 µm diameter; Ernster & Schatz, 1981; Wrigglesworth, Packer, & Branton, 1970) or extracellular DNA, rather than cell or tissue DNA. One possible explanation is that microbial activity increases as water temperature increases, allowing degradation of mitochondrial double cell membranes and the mitochondrial DNA within. Furthermore, such a reduction of eDNA size with higher temperature might contribute to the water temperature‐dependent degradation of eDNA. For example, the nominal pore size of the GF/F filter, which we used for estimation of eDNA decay rates, was 0.7 μm, which means that the filter cannot capture eDNA smaller than 0.7 μm. A decrease in the amount of eDNA larger than the filter pore size as temperature increased might result in such water temperature‐dependent degradation of eDNA.

The results showed that the most abundant size fraction was 3–10 μm for the Medium fish size, 0.8–3 μm for the Small fish size, and 0.4–0.8 μm for the Large fish size. The percentage of eDNA at the >10 μm size fraction was not significantly, but statistically marginally different among fish biomass levels (*p = *0.0862; Figure 3). Such differences might partly reflect the effect of fish density. For example, the percentage of eDNA at 0.4–0.8 µm was larger for Large size level than for other size levels, which might be caused by the increase in microbial activity due to the increase in fish biomass in the tank. In addition, this result might suggest that the eDNA origin, state, and their component ratio could vary depending on fish biomass or, possibly, their development stage. Further study would be needed to clarify the relationships between the developmental stage and aforementioned eDNA characteristics.

The results showed that eDNA size distribution varied temporally (Figure 4). At first, the percentage of eDNA at >10 µm size fraction dramatically increased just after the fish removal. Considering that such handling stress could cause the fish to shed large sized DNA, such as their scale and mucus (Merkes et al., 2014; Sassoubre et al., 2016), this could be reasonable. In addition, the percentages of eDNA at small size fractions increased with a time passage and that at >10 µm decrease. These temporal shifts in eDNA size distribution to smaller size fractions might represent the dynamics of eDNA described above. These results demonstrated that the states of eDNA changed with time passage after it is released from organisms. Further study would be needed for the relationship between the persistence of eDNA and its state (i.e., intra/extracellular and within live/dead cells).

## 5 CONCLUSION

In conclusion, water temperature and fish biomass facilitated eDNA shedding and degradation. The higher eDNA decay rates with larger biomass could reflect the activity and abundance of microbes and extra‐organism nucleases in the water, and the higher eDNA shedding rates with higher temperature might due to higher metabolism and physiological activity of organisms. In addition, eDNA size distribution also varied depending on water temperature, fish biomass, and time passage. The increases of smaller sized fractions of eDNA with higher temperature and the difference in eDNA size distribution among fish biomass might reflect the microbial activity in the water. Furthermore, the temporal changes of eDNA size distribution showed that the state of eDNA could vary with time passage due to degradation caused by various environmental factors after release into the environment.

Although this study clarified some of the eDNA dynamics, the research area needs further study. For example, although our findings imply that microbes and extra‐organism nucleases are involved in eDNA degradation, and that metabolism affects the eDNA shedding rate, these aspects were not demonstrated directly in this study. In addition, we could not assess the effect of seasonal change in the seawater (e.g., nutrient load, salinity, chlorophyll) despite our experimental periods over different season (Supporting Information Table S1). There is therefore a possibility that certain chemical and microbial conditions could influence the behavior of fish individuals as well as that of eDNA, and these could be subjects of future studies. Moreover, there has been little research on the physiological source of eDNA production and the physical aspects of eDNA such as its structure and length (Barnes & Turner, 2016). A greater understanding and accumulation of basic information on eDNA would improve eDNA analysis and enable researchers to maximize the potential of future eDNA applications. This study would lay a groundwork that can be used in further eDNA research.

## CONFLICT OF INTEREST

None declared.

## AUTHOR CONTRIBUTIONS

T.J., H.M., R.M., and T.M. conceived and designed the experiments. T.J., H.M., and S.Y. performed tank experiments. T.J. analyzed the data. T.J., H.M., S.Y., R.M., and T.M. wrote and edited the manuscript.

## Data Availability

All data, including the raw values for the qPCR experiments, are included in the supporting information.

## References

[ece34802-bib-0001] Balasingham, K. D. , Walter, R. P. , Mandrak, N. E. , & Heath, D. D. (2017). Environmental DNA detection of rare and invasive fish species in two great lakes tributaries. Molecular Ecology, 27(1), 112–127. 10.1111/mec.14395 29087006

[ece34802-bib-0002] Barnes, M. A. , & Turner, C. R. (2016). The ecology of environmental DNA and implications for conservation genetics. Conservation Genetics, 17(1), 1–17. 10.1007/s10592-015-0775-4

[ece34802-bib-0003] Barnes, M. A. , Turner, C. R. , Jerde, C. L. , Renshaw, M. A. , Chadderton, W. L. , & Lodge, D. M. (2014). Environmental conditions influence eDNA persistence in aquatic systems. Environmental Science & Technology, 48(3), 1819–1827. 10.1021/es404734p 24422450

[ece34802-bib-0004] Barton, B. A. (2002). Stress in fishes: A diversity of responses with particular reference to changes in circulating corticosteroids. Integrative and Comparative Biology, 42(3), 517–525. 10.1093/icb/42.3.517 21708747

[ece34802-bib-0005] Bergmann, C. (1847). Über die verhältnisse der wärmeökonomie der thiere zu ihrer grösse. Göttinger Studien, 5, 595–708.

[ece34802-bib-0006] Bista, I. , Carvalho, G. R. , Walsh, K. , Seymour, M. , Hajibabaei, M. , Lallias, D. , … Creer, S. (2017). Annual time‐series analysis of aqueous eDNA reveals ecologically relevant dynamics of lake ecosystem biodiversity. Nature Communications, 8, 14087 10.1038/ncomms14087 PMC5253663 28098255

[ece34802-bib-0007] Boussarie, G. , Bakker, J. , Wangensteen, O. S. , Mariani, S. , Bonnin, L. , Juhel, J. B. , … David, M. (2018). Environmental DNA illuminates the dark diversity of sharks. Science. Advances, 4(5), eaap9661 10.1126/sciadv.aap9661 PMC5931749 29732403

[ece34802-bib-0008] Bylemans, J. , Furlan, E. M. , Gleeson, D. M. , Hardy, C. M. , & Duncan, R. P. (2018). Does size matter? An experimental evaluation of the relative abundance and decay rates of aquatic eDNA. Environmental Science & Technology, 52(11), 6408–6416. 29757618 10.1021/acs.est.8b01071

[ece34802-bib-0009] Bylemans, J. , Furlan, E. M. , Hardy, C. M. , McGuffie, P. , Lintermans, M. , & Gleeson, D. M. (2017). An environmental DNA-based method for monitoring spawning activity: A case study, using the endangered Macquarie perch (*Macquaria australasica*). Methods in Ecology and Evolution, 8(5), 646–655.

[ece34802-bib-0010] Clarke, A. , & Johnston, N. M. (1999). Scaling of metabolic rate with body mass and temperature in teleost fish. Journal of Animal Ecology, 68(5), 893–905. 10.1046/j.1365-2656.1999.00337.x

[ece34802-bib-0011] Cook, J. R. , & Heyse, J. F. (2000). Use of an angular transformation for ratio estimation in cost‐effectiveness analysis. Statistics in Medicine, 19(21), 2989–3003. 11042628 10.1002/1097-0258(20001115)19:21<2989::aid-sim599>3.0.co;2-g

[ece34802-bib-0012] Corinaldesi, C. , Beolchini, F. , & Dell’Anno, A. (2008). Damage and degradation rates of extracellular DNA in marine sediments: Implications for the preservation of gene sequences. Molecular Ecology, 17(17), 3939–3951. 10.1111/j.1365-294X.2008.03880.x 18643876

[ece34802-bib-0013] Deiner, K. , Fronhofer, E. A. , Mächler, E. , Walser, J. C. , & Altermatt, F. (2016). Environmental DNA reveals that rivers are conveyer belts of biodiversity information. Nature Communications, 7, 12544 10.1038/ncomms12544 PMC5013555 27572523

[ece34802-bib-0014] Dell'Anno, A. , & Corinaldesi, C. (2004). Degradation and turnover of extracellular DNA in marine sediments: Ecological and methodological considerations. Applied and Environmental Microbiology, 70(7), 4384–4386. 10.1128/AEM.70.7.4384-4386.2004 15240325 PMC444808

[ece34802-bib-0015] Díaz‐Ferguson, E. E. , & Moyer, G. R. (2014). History, applications, methodological issues and perspectives for the use environmental DNA (eDNA) in marine and freshwater environments. Revista De Biologia Tropical, 62, 1273–1284. 10.15517/rbt.v62i4.13231 25720166

[ece34802-bib-0016] Doi, H. , Inui, R. , Akamatsu, Y. , Kanno, K. , Yamanaka, H. , Takahara, T. , & Minamoto, T. (2016). Environmental DNA analysis for estimating the abundance and biomass of stream fish. Freshwater Biology, 1(62), 30–39.

[ece34802-bib-0017] Doi, H. , Uchii, K. , Takahara, T. , Matsuhashi, S. , Yamanaka, H. , & Minamoto, T. (2015). Use of droplet digital PCR for estimation of fish abundance and biomass in environmental DNA surveys. PLoS ONE, 10(3), e0122763 10.1371/journal.pone.0122763 25799582 PMC4370432

[ece34802-bib-0018] Dunn, N. , Priestley, V. , Herraiz, A. , Arnold, R. , & Savolainen, V. (2017). Behavior and season affect crayfish detection and density inference using environmental DNA. Ecology and Evolution, 7(19), 7777–7785. 10.1002/ece3.3316 29043033 PMC5632632

[ece34802-bib-0019] Eichmiller, J. J. , Best, S. E. , & Sorensen, P. W. (2016). Effects of temperature and trophic state on degradation of environmental DNA in lake water. Environmental Science & Technology, 50(4), 1859–1867. 10.1021/acs.est.5b05672 26771292

[ece34802-bib-0020] Ellison, S. L. , English, C. A. , Burns, M. J. , & Keer, J. T. (2006). Routes to improving the reliability of low level DNA analysis using real‐time PCR. BMC Biotechnology, 6(1), 33. 16824215 10.1186/1472-6750-6-33 PMC1559608

[ece34802-bib-0021] Ernster, L. , & Schatz, G. (1981). Mitochondria: A historical review. The Journal of Cell Biology, 91(3), 227s–255s. 10.1083/jcb.91.3.227s 7033239 PMC2112799

[ece34802-bib-0022] Ficetola, G. F. , Miaud, C. , Pompanon, F. , & Taberlet, P. (2008). Species detection using environmental DNA from water samples. Biology Letters, 4(4), 423–425. 10.1098/rsbl.2008.0118 18400683 PMC2610135

[ece34802-bib-0023] Fukumoto, S. , Ushimaru, A. , & Minamoto, T. (2015). A basin‐scale application of environmental DNA assessment for rare endemic species and closely related exotic species in rivers: A case study of giant salamanders in Japan. Journal of Applied Ecology, 52(2), 358–365. 10.1111/1365-2664.12392

[ece34802-bib-0024] Goldberg, C. S. , Strickler, K. M. , & Pilliod, D. S. (2015). Moving environmental DNA methods from concept to practice for monitoring aquatic macroorganisms. Biological Conservation, 183, 1–3. 10.1016/j.biocon.2014.11.040

[ece34802-bib-0025] Hansen, B. K. , Bekkevold, D. , Clausen, L. W. , & Nielsen, E. E. (2018). The sceptical optimist: Challenges and perspectives for the application of environmental DNA in marine fisheries. Fish and Fisheries, 19(5), 751–768. 10.1111/faf.12286

[ece34802-bib-0027] Jo, T. , Murakami, H. , Masuda, R. , Sakata, M. , Yamamoto, S. , & Minamoto, T. (2017). Rapid degradation of longer DNA fragments enables the improved estimation of distribution and biomass using environmental DNA. Molecular Ecology Resources, 17(6), e25–e33. 10.1111/1755-0998.12685 28449215

[ece34802-bib-0028] Klymus, K. E. , Richter, C. A. , Chapman, D. C. , & Paukert, C. (2015). Quantification of eDNA shedding rates from invasive bighead carp *Hypophthalmichthys nobilis* and silver carp *Hypophthalmichthys molitrix* . Biological Conservation, 183, 77–84. 10.1016/j.biocon.2014.11.020

[ece34802-bib-0029] Lacoursière‐Roussel, A. , Howland, K. , Normandeau, E. , Grey, E. K. , Archambault, P. , Deiner, K. , … Bernatchez, L. (2018). eDNA metabarcoding as a new surveillance approach for coastal Arctic biodiversity. Ecology & Evolution, 8(16), 7763–7777. 10.1002/ece3.4213 30250661 PMC6144963

[ece34802-bib-0030] Lance, R. F. , Klymus, K. E. , Richter, C. A. , Guan, X. , Farrington, H. L. , Carr, M. R. , … Baerwaldt, K. L. (2017). Experimental observations on the decay of environmental DNA from bighead and silver carps. Management of Biological Invasions, 8(3), 343–359. 10.3391/mbi.2017.8.3.08

[ece34802-bib-0031] Levy‐Booth, D. J. , Campbell, R. G. , Gulden, R. H. , Hart, M. M. , Powell, J. R. , Klironomos, J. N. , … Dunfield, K. E. (2007). Cycling of extracellular DNA in the soil environment. Soil Biology and Biochemistry, 39(12), 2977–2991. 10.1016/j.soilbio.2007.06.020

[ece34802-bib-0032] Lindahl, T. (1993). Instability and decay of the primary structure of DNA. Nature, 362(6422), 709–715. 8469282 10.1038/362709a0

[ece34802-bib-0033] Lodge, D. M. , Turner, C. R. , Jerde, C. L. , Barnes, M. A. , Chadderton, L. , Egan, S. P. , … Pfrender, M. E. (2012). Conservation in a cup of water: Estimating biodiversity and population abundance from environmental DNA. Molecular Ecology, 21(11), 2555–2558. 10.1111/j.1365-294X.2012.05600.x 22624944 PMC3412215

[ece34802-bib-0034] Martellini, A. , Payment, P. , & Villemur, R. (2005). Use of eukaryotic mitochondrial DNA to differentiate human, bovine, porcine and ovine sources in fecally contaminated surface water. Water Research, 39(4), 541–548. 10.1016/j.watres.2004.11.012 15707626

[ece34802-bib-0035] Maruyama, A. , Nakamura, K. , Yamanaka, H. , Kondoh, M. , & Minamoto, T. (2014). The release rate of environmental DNA from juvenile and adult fish. PLoS ONE, 9(12), e114639 10.1371/journal.pone.0114639 25479160 PMC4257714

[ece34802-bib-0036] Masuda, R. (2008). Seasonal and interannual variation of subtidal fish assemblages in Wakasa Bay with reference to the warming trend in the Sea of Japan. Environmental Biology of Fishes, 82, 387–399.

[ece34802-bib-0038] Merkes, C. M. , McCalla, S. G. , Jensen, N. R. , Gaikowski, M. P. , & Amberg, J. J. (2014). Persistence of DNA in carcasses, slime and avian feces may affect interpretation of environmental DNA data. PLoS ONE, 9(11), e113346 10.1371/journal.pone.0113346 25402206 PMC4234652

[ece34802-bib-0039] Minamoto, T. , Yamanaka, H. , Takahara, T. , Honjo, M. N. , & Kawabata, Z. (2012). Surveillance of fish species composition using environmental DNA. Limnology, 13(2), 193–197. 10.1007/s10201-011-0362-4

[ece34802-bib-0040] Minamoto, T. , Fukuda, M. , Katsuhara, K. R. , Fujiwara, A. , Hidaka, S. , Yamamoto, S. , … Masuda, R. (2017). Environmental DNA reflects spatial and temporal jellyfish distribution. PLoS ONE, 12(2), e0173073 10.1371/journal.pone.0173073 28245277 PMC5330514

[ece34802-bib-0041] Morita, K. , Fukuwaka, M. A. , Tanimata, N. , & Yamamura, O. (2010). Size‐dependent thermal preferences in a pelagic fish. Oikos, 119(8), 1265–1272. 10.1111/j.1600-0706.2009.18125.x

[ece34802-bib-0042] Nakamura, T. , & Hamano, A. (2009). Seasonal differences in the vertical distribution pattern of Japanese jack mackerel, *Trachurus japonicus*: Changes according to age? ICES Journal of Marine Science, 66(6), 1289–1295. 10.1093/icesjms/fsp114

[ece34802-bib-0043] Nielsen, K. M. , Johnsen, P. J. , Bensasson, D. , & Daffonchio, D. (2007). Release and persistence of extracellular DNA in the environment. Environmental Biosafety Research, 6(1–2), 37–53. 10.1051/ebr:2007031 17961479

[ece34802-bib-0044] Person‐Le Ruyet, J. , Mahe, K. , Le Bayon, N. , & Le Delliou, H. (2004). Effects of temperature on growth and metabolism in a Mediterranean population of European sea bass, *Dicentrarchus labrax* . Aquaculture, 237(1), 269–280. 10.1016/j.aquaculture.2004.04.021

[ece34802-bib-0045] Pilliod, D. S. , Goldberg, C. S. , Arkle, R. S. , & Waits, L. P. (2014). Factors influencing detection of eDNA from a stream‐dwelling amphibian. Molecular Ecology Resources, 14(1), 109–116. 10.1111/1755-0998.12159 24034561

[ece34802-bib-0046] Poté, J. , Ackermann, R. , & Wildi, W. (2009). Plant leaf mass loss and DNA release in freshwater sediments. Ecotoxicology and Environmental Safety, 72(5), 1378–1383. 10.1016/j.ecoenv.2009.04.010 19419763

[ece34802-bib-0047] R Core Team (2016). R: A language and environment for statistical computing. Vienna, Austria: R Foundation for Statistical Computing.

[ece34802-bib-0048] Sandersfeld, T. , Mark, F. C. , & Knust, R. (2017). Temperature‐dependent metabolism in Antarctic fish: Do habitat temperature conditions affect thermal tolerance ranges? Polar Biology, 40(1), 141–149. 10.1007/s00300-016-1934-x

[ece34802-bib-0049] Sansom, B. J. , & Sassoubre, L. M. (2017). Environmental DNA (eDNA) shedding and decay rates to model freshwater mussel eDNA transport in a river. Environmental Science & Technology, 51(24), 14244–14253. 10.1021/acs.est.7b05199 29131600

[ece34802-bib-0050] Sassoubre, L. M. , Yamahara, K. M. , Gardner, L. D. , Block, B. A. , & Boehm, A. B. (2016). Quantification of environmental DNA (eDNA) shedding and decay rates for three marine fish. Environmental Science & Technology, 50(19), 10456–10464. 10.1021/acs.est.6b03114 27580258

[ece34802-bib-0051] Sigsgaard, E. E. , Nielsen, I. B. , Bach, S. S. , Lorenzen, E. D. , Robinson, D. P. , Knudsen, S. W. , … Thomsen, P. F. (2016). Population characteristics of a large whale shark aggregation inferred from seawater environmental DNA. Nature Ecology & Evolution, 1, 0004 10.1038/s41559-016-0004 28812572

[ece34802-bib-0052] Strickler, K. M. , Fremier, A. K. , & Goldberg, C. S. (2015). Quantifying effects of UV‐B, temperature, and pH on eDNA degradation in aquatic microcosms. Biological Conservation, 183, 85–92. 10.1016/j.biocon.2014.11.038

[ece34802-bib-0053] Taberlet, P. , Coissac, E. , Hajibabaei, M. , & Rieseberg, L. H. (2012). Environmental DNA. Molecular Ecology, 21(8), 1789–1793. 10.1111/j.1365-294X.2012.05542.x 22486819

[ece34802-bib-0054] Takahara, T. , Honjo, M. N. , Uchii, K. , Minamoto, T. , Doi, H. , Ito, T. , & Kawabata, Z. (2014). Effects of daily temperature fluctuation on the survival of carp infected with Cyprinid herpesvirus 3. Aquaculture, 433, 208–213. 10.1016/j.aquaculture.2014.06.001

[ece34802-bib-0055] Takahara, T. , Minamoto, T. , Yamanaka, H. , Doi, H. , & Kawabata, Z. (2012). Estimation of fish biomass using environmental DNA. PLoS ONE, 7(4), e35868 10.1371/journal.pone.0035868 22563411 PMC3338542

[ece34802-bib-0056] Thomsen, P. F. , Kielgast, J. , Iversen, L. L. , Møller, P. R. , Rasmussen, M. , & Willerslev, E. (2012). Detection of a diverse marine fish fauna using environmental DNA from seawater samples. PLoS ONE, 7(8), e41732 10.1371/journal.pone.0041732 22952584 PMC3430657

[ece34802-bib-0057] Thomsen, P. F. , & Willerslev, E. (2015). Environmental DNA – An emerging tool in conservation for monitoring past and present biodiversity. Biological Conservation, 183, 4–18. 10.1016/j.biocon.2014.11.019

[ece34802-bib-0058] Thomsen, P. F. , Kielgast, J. O. S. , Iversen, L. L. , Wiuf, C. , Rasmussen, M. , Gilbert, M. T. P. , … Willerslev, E. (2012). Monitoring endangered freshwater biodiversity using environmental DNA. Molecular Ecology, 21(11), 2565–2573. 10.1111/j.1365-294X.2011.05418.x 22151771

[ece34802-bib-0059] Tsuji, S. , Ushio, M. , Sakurai, S. , Minamoto, T. , & Yamanaka, H. (2017). Water temperature‐dependent degradation of environmental DNA and its relation to bacterial abundance. PLoS ONE, 12(4), e0176608 10.1371/journal.pone.0176608 28448613 PMC5407774

[ece34802-bib-0060] Tsuji, S. , Yamanaka, H. , & Minamoto, T. (2016). Effects of water pH and proteinase K treatment on the yield of environmental DNA from water samples. Limnology, 18(1), 1–7. 10.1007/s10201-016-0483-x

[ece34802-bib-0061] Turner, C. R. , Barnes, M. A. , Xu, C. C. , Jones, S. E. , Jerde, C. L. , & Lodge, D. M. (2014). Particle size distribution and optimal capture of aqueous macrobial eDNA. Methods in Ecology and Evolution, 5(7), 676–684. 10.1111/2041-210X.12206

[ece34802-bib-0062] Turner, C. R. , Uy, K. L. , & Everhart, R. C. (2015). Fish environmental DNA is more concentrated in aquatic sediments than surface water. Biological Conservation, 183, 93–102. 10.1016/j.biocon.2014.11.017

[ece34802-bib-0063] Wilcox, T. M. , McKelvey, K. S. , Young, M. K. , Lowe, W. H. , & Schwartz, M. K. (2015). Environmental DNA particle size distribution from Brook Trout (*Salvelinus fontinalis*). Conservation Genetics Resources, 7(3), 639–641. 10.1007/s12686-015-0465-z

[ece34802-bib-0064] Wrigglesworth, J. M. , Packer, L. , & Branton, D. (1970). Organization of mitochondrial structure as revealed by freeze‐etching. Biochimica Et Biophysica Acta (BBA) ‐ Bioenergetics, 205(2), 125–135. 10.1016/0005-2728(70)90243-4 4192700

[ece34802-bib-0065] Yamamoto, S. , Masuda, R. , Sato, Y. , Sado, T. , Araki, H. , Kondoh, M. , … Miya, M. (2017). Environmental DNA metabarcoding reveals local fish communities in a species‐rich coastal sea. Scientific Reports, 7, 40368 10.1038/srep40368 28079122 PMC5227697

[ece34802-bib-0066] Yamamoto, S. , Minami, K. , Fukaya, K. , Takahashi, K. , Sawada, H. , Murakami, H. , … Kondoh, M. (2016). Environmental DNA as a ‘snapshot’ of fish distribution: A case study of Japanese jack mackerel in Maizuru Bay, Sea of Japan. PLoS ONE, 11(3), e0149786 10.1371/journal.pone.0149786 26933889 PMC4775019

[ece34802-bib-0067] Yamanaka, H. , & Minamoto, T. (2016). The use of environmental DNA of fishes as an efficient method of determining habitat connectivity. Ecological Indicators, 62, 147–153. 10.1016/j.ecolind.2015.11.022

