# Supplementary material for: Effect of water temperature and fish biomass on environmental DNA shedding, degradation, and size distribution
Source: Ecol Evol. 2019 Jan 21;9(3):1135–46. doi: 10.1002/ece3.4802 (PMC6374661; doi:10.1002/ece3.4802)

Fig. S1. Diagram showing eDNA sampling for the estimation of eDNA size distribution. Targeting all fish biomass levels, we filtered water samples only at time bfr using a series of polycarbonate membrane filters with 10, 3, 0.8, and 0.4 µm pore size. Besides, targeting Small and Large fish biomass levels, we temporally filtered water samples at time bfr, 0, 6, 12, 18 using same filters with 10, 3, 0.8, and 0.2 µm pore size.

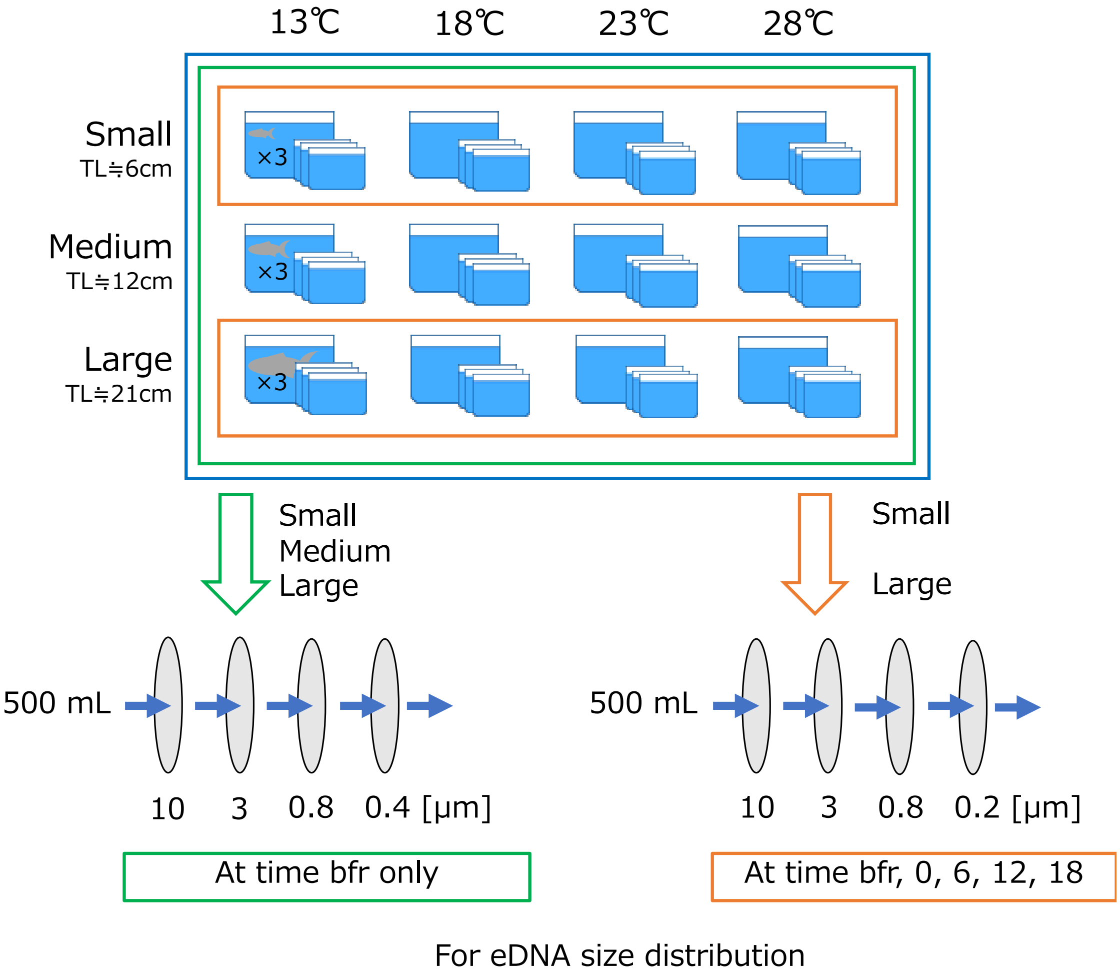

Supplement: Supplementary file 1 [file ECE3-9-1135-s001.pdf]
